# Plasma matrix metalloproteinases are associated with incident cardiovascular disease and all-cause mortality in patients with type 1 diabetes: a 12-year follow-up study

**DOI:** 10.1186/s12933-017-0539-1

**Published:** 2017-04-26

**Authors:** S. A. Peeters, L. Engelen, J. Buijs, A. Jorsal, H.-H. Parving, L. Tarnow, P. Rossing, C. G. Schalkwijk, C. D. A. Stehouwer

**Affiliations:** 1 Department of Internal Medicine, Maastricht University Medical Centre, P.O. Box 5800, 6202 AZ Maastricht, The Netherlands; 2 Department of Internal Medicine, Zuyderland hospital, Heerlen, The Netherlands; 3 CARIM School for Cardiovascular Diseases, Maastricht University Medical Centre, Maastricht, The Netherlands; 4 Department of Cardiology, Aarhus University Hospital, Aarhus, Denmark; 5 Steno Diabetes Center, Gentofte, Denmark; 6 Department of Medical Endocrinology, Rigshospitalet, Copenhagen, Denmark; 7 Faculty of Health Science, Aarhus University, Aarhus, Denmark; 8 Faculty of Health, University of Copenhagen, Copenhagen, Denmark; 9 Nordsjaellands Hospital, Hilleroed, Denmark

**Keywords:** Type 1 diabetes, Cardiovascular disease, All-cause mortality, Matrix metalloproteinase, Tissue inhibitor of metalloproteinase, Estimated glomerular filtration rate

## Abstract

**Background:**

Altered regulation of extracellular matrix remodeling by matrix metalloproteinases (MMPs) and tissue inhibitor of metalloproteinase (TIMP) may contribute to vascular complications in type 1 diabetes. We investigated associations between plasma MMP-1, -2, -3, -9, -10 and TIMP-1, and cardiovascular events and all-cause mortality in type 1 diabetic patients.

**Methods:**

We prospectively followed 337 type 1 diabetic patients [mean age 41.4 years (9.6), 39% female], 170 with and 167 without diabetic nephropathy, with median follow-up of 12.3 years. Survival analyses were applied to investigate differences in plasma MMP-1, -2, -3, -9, -10, and TIMP-1-levels in patients with and without a cardiovascular event and in those who died vs survivors. All analyses were adjusted for age, sex, duration of diabetes, HbA1c, nephropathy and for other conventional cardiovascular risk factors.

**Results:**

After adjustment for potential confounders, higher MMP-2 plasma levels were significantly associated with higher incidence of cardiovascular events [HR 1.49 (95% CI 1.11; 1.99)], and higher plasma levels of MMP-1 [1.38 (1.07; 1.78)], MMP-2 [1.60 (1.19; 2.15)] and MMP-3 [1.39 (1.05; 1.85)] were associated with all-cause mortality. All associations were independent of low-grade inflammation and endothelial dysfunction as estimated by plasma markers. Associations between MMP-2 and cardiovascular events and between MMP-3 and mortality were attenuated after further adjustment for eGFR and changes in eGFR.

**Conclusions:**

Higher levels of MMP-2 are associated with CVD and higher MMP-1, -2 and -3 with all-cause mortality. In addition, associations between MMP-2 and CVD, and MMP-3 and mortality were attenuated after adjustment for eGFR while both MMPs were associated with eGFR decline, indicating a possible mediating role of eGFR.

**Electronic supplementary material:**

The online version of this article (doi:10.1186/s12933-017-0539-1) contains supplementary material, which is available to authorized users.

## Background

The pathophysiological mechanisms leading to cardiovascular disease (CVD) in type 1 diabetes [1] have only been partly elucidated. Recent data suggest that altered regulation of extracellular matrix (ECM) remodeling by matrix metalloproteinases (MMPs) could play a role [2]. MMPs are proteases, which degrade ECM components in normal and pathological conditions. Increased plasma MMP-2, for example, has been associated with macrovascular disease by increasing vascular remodeling as well as thrombus formation [3, 4]. MMP production may be enhanced by hyperglycemia, pro-inflammatory mediators, reactive oxygen species and aldosterone [3]; MMP activity is dependent on the number of MMPs and their inhibitors, such as tissue inhibitor of metalloproteinase (TIMP) and α2-macroglobulin [5]. In turn, increased activity of MMP-2 and MMP-9 is associated with low-grade inflammation (LGI) [6] and endothelial dysfunction (ED) [7], and increased MMP-9 levels have been associated with renal dysfunction [8]. Therefore, LGI, ED and renal dysfunction constitute potential pathophysiological mechanisms through which MMPs could lead to CVD.

Interestingly, higher plasma levels of MMP-2 and -10 have been observed in patients with type 1 diabetes compared to non-diabetic controls [9, 10]. In cross-sectional studies, individuals with type 1 diabetes and retinopathy or nephropathy showed significantly higher circulating levels of MMP-9 and -10 compared to individuals without these complications [10, 11]. These data support the concept that MMPs may play an important role in the development of CVD. In addition, several prospective studies in (mainly) non-diabetic individuals have shown independent associations between plasma MMP-2 [12], MMP-9 [13] and TIMP-1 [14] and CVD, as well as associations between MMP-1 [15], MMP-2 [12, 16, 17] and -3 [18, 19] and all-cause mortality. However, to our knowledge, no prospective studies have been reported on the associations between plasma MMPs or TIMP levels and CVD or all-cause mortality in type 1 diabetes.

In view of these considerations, we investigated associations, in type 1 diabetes, between plasma MMP-1, -2, -3, -9 and -10, and TIMP-1 and incident non-fatal and fatal cardiovascular events, as well as all-cause mortality, in a prospective study with more than 12 years of follow-up. In addition, we investigated the extent to which LGI, ED and renal dysfunction contributed to these associations.

## Methods

### Study population and design

In 1993, 199 type 1 diabetic patients with diabetic nephropathy and older than 18 years were enrolled in a prospective observational study at the outpatient clinic at Steno Diabetes Center. Diabetic nephropathy was defined as persistent macroalbuminuric [urinary albumin excretion (UAE) >300 mg/24 h] in at least two out of three previous consecutive 24-h urine collections, presence of retinopathy, and absence of other kidney or urinary tract disease. In addition, 192 normoalbuminuric diabetic patients (UAE <30 mg/24 h), matched for age, sex, and duration of diabetes, were enrolled for comparison [20].

Patients with prior CVD (n = 24), who were lost to follow-up (n = 17), with end-stage renal disease at baseline (n = 7) and those with missing data on MMPs/TIMP-1 (n = 6) were excluded from the present analysis resulting in a total study population of 337 individuals. Patient selection and inclusion criteria have been described in detail elsewhere [21]. The study was approved by the local ethics committee, in accordance with the Helsinki Declaration, and all patients gave their written informed consent.

### Baseline investigations

Commercially available ELISA kits were used to measure levels of MMP-1, MMP-2, MMP-3, MMP-9, MMP-10 and TIMP-1 in EDTA plasma samples [Human MMP 3-Plex Kit (for MMP-1, -3 and -9), Human MMP-2-Plex Kit (for MMP-2 and -10) and Human TIMP-1 Kit, MSD, Rockville, USA], according to the manufacturer’s protocol. The MMPs were detected in both pro- and active form. TIMP-1 was detected in the active form. The intra- and inter-assay coefficients of variation were 6.6 and 9.7% for MMP-1, 3.7 and 7.1% for MMP-2, 7.2 and 14.1% for MMP-3, 4.6 and 13.7% for MMP-9, 2.9 and 7.6% for MMP-10, and 5.1 and 7.4% for TIMP-1, respectively.

### Other baseline measurements

Measurements of other biomarkers and risk factors have been described in detail elsewhere [20]. All clinical investigations were performed in the morning after an overnight fast. In 88% of the normoalbuminuric and 24% of macroalbuminuric patients antihypertensive medication was never prescribed. Patients using antihypertensive medication were asked to stop their antihypertensive and diuretic treatment eight days before the examination, but 15% of the 337 patients (27% in the nephropathy group) considered in our analyses did not comply with this recommendation.

Arterial blood pressure was measured twice, with an appropriate cuff size, following at least a 10-min rest in supine position. Mean arterial pressure (MAP) was calculated as [systolic blood pressure + (2*diastolic blood pressure)]/3. Body mass index (BMI) was defined as weight (kg) divided by height (m) squared. An ELISA was used to measure UAE from 24-h urine collections. Serum creatinine was determined by a kinetic Jaffé method and we estimated the glomerular filtration rate (eGFR) using the chronic kidney disease epidemiology Collaboration (CKD-EPI) equation [22], after creatinine was IDMS standardized [23]. Patients were divided into three groups according to their smoking status: never, former or current smokers.

An in-house ELISA was used to determine high-sensitivity C-reactive protein (CRP) [24] and commercially available ELISA kits were used to measure plasma levels of secreted phospholipase A2 (sPLA2), interleukin-6 (IL-6), soluble vascular cell adhesion molecule-1 (sVCAM-1) and soluble intercellular adhesion molecule-1 (sICAM-1).

### Follow-up and study end-points

All patients were followed up to the last visit at Steno Diabetes Center, until September 1st 2006, death (n = 83) or emigration (n = 3). All patients were traced through the national register during autumn 2006. When a patient died before 1 September 2006, the date of death was recorded and the primary cause of death was obtained from the death certificate, which was reviewed by two independent observers. Additionally, information from necropsy reports was included, when available. All deaths were classified as cardiovascular unless an unequivocal non-cardiovascular cause was established. At the end of follow up, non-fatal events were retrieved from patients’ records at Steno Diabetes Center or other hospital records. The primary endpoint of this study was fatal or non-fatal CVD (i.e. myocardial infarction, percutaneous coronary intervention, coronary bypass grafting, amputation due to ischemia, vascular surgery for peripheral atherosclerotic disease and stroke). Ischemic heart disease was examined through a WHO questionnaire combined with an ECG [20]. In several patients, who were alive at follow-up and had more than one cardiovascular event during follow-up, the first event was used in the analyses. All-cause mortality was the secondary end-point.

### Statistical analyses

All analyses were performed with SPSS version 20 for Windows (SPSS, Chicago, IL, USA). Triglycerides, UAE, CRP, IL-6, sPLA2, MMP-1, MMP-2, MMP-3, MMP-9, MMP-10 and TIMP-1 showed a skewed distribution and were log_℮_ transformed prior to further analyses. Student’s t or Chi square tests were performed for comparisons of baseline characteristics between groups, when appropriate. Cox proportional hazards regression models were applied to investigate associations between lnMMP-1, lnMMP-2, lnMMP-3, lnMMP-9, lnMMP-10 and lnTIMP-1 and study endpoints with adjustment for sex, age, duration of diabetes, HbA1c and nephropathy-no nephropathy status (model 1). Further adjustments were performed for BMI, MAP, total cholesterol, smoking status, antihypertensive treatment and whether or not patients withheld their medication prior to baseline examinations (model 2). We used z-scores (in SD) of lnMMPs and lnTIMP-1 to enable direct comparisons between the strengths of the associations (if any) between the various markers and outcome measures.

An LGI score was calculated as the z-score of the average of the z-scores of lnIL-6, lnCRP, sICAM-1, and lnsPLA2 [2, 25–27]. Similarly, an ED score was calculated as the z-score of the average of the z-scores of sICAM-1 and sVCAM-1 [2, 26, 27]. We examined the cross-sectional associations between lnMMP-1, lnMMP-2, lnMMP-3, lnMMP-9, lnMMP-10 and lnTIMP-1, and renal function (i.e. eGFR and ln-UAE), the LGI score and the ED score using linear regression analyses. Further analyses with adjustment of the associations between MMPs and TIMP-1 and study endpoints for eGFR, and ln-UAE (model 3), LGI (model 4) and ED (model 5) were performed to evaluate the extent to which these pathophysiological processes could explain the associations between MMPs/TIMP-1 and study outcomes. Additionally, in case renal function does explain part of the association between circulating MMPs and TIMP-1 and outcome measures, the possible additional influence of decrease in eGFR over time will also be investigated. Finally, we investigated whether or not any such associations differed between patients with and without nephropathy or between men and women by adding interaction terms between nephropathy or sex and MMPs or TIMP-1 to the models. Whenever a p value <0.1 was observed, results were presented for both groups separately.

## Results

Additional file 1: Figure S1 depicts the flowchart concerning patient selection. During a median follow-up of 12.3 years (interquartile range 7.7–12.5), 86 patients had a non-fatal (n = 54) or fatal (n = 49) cardiovascular event. Eighty-three patients died from various causes: cardiovascular (n = 49), diabetes (n = 4), infection (n = 13), cancer (n = 6), suicide (n = 4) and other (n = 7). Patients with a cardiovascular event or who died during follow-up were significantly older, had a longer duration of diabetes, higher levels of HbA1c and a higher prevalence of other cardiovascular risk factors (Table 1). Additionally, MMP-1, MMP-2, MMP-3, MMP-9, MMP-10 and TIMP-1 were higher in this group compared to patients without cardiovascular events or surviving the period of follow-up. Additional file 1: Table S1 shows the baseline characteristics according to nephropathy-no nephropathy status. Additional file 1: Table S2 shows the correlations between five MMPs and TIMP-1.

**Table 1:** Baseline characteristics according to the incidence of a cardiovascular event or death during follow-up

	CVD event	No CVD event	p value	Patients dead at follow-up n = 83	Patients alive at follow-up n = 254	p value
	n = 86	n = 251				
Age (years)	44.5 (9.0)	40.4 (9.6)	0.001	45.3 (10.0)	40.2 (9.2)	<0.001
Sex: male/female (%)	62/38	60/40	0.81	69/31	58/42	0.081
Duration of diabetes (years)	30.7 (8.7)	26.7 (7.5)	<0.001	30.2 (9.9)	26.9 (7.1)	0.006
HbA1c (%)	9.5 (1.5)	8.9 (1.4)	<0.001	9.6 (1.5)	8.8 (1.4)	<0.001
HbA1c (mmol/mol)	80.5 (16.3)	73.4 (15.0)	<0.001	81.6 (16.5)	73.1 (14.8)	<0.001
Nephropathy (%)	76	42	<0.001	78	41	<0.001
Retinopathy (no/simplex/proliferative) (%)	8/37/55	20/45/35	0.002	6/37/57	21/45/34	<0.001
BMI (kg/m^2^)	23.9 (3.3)	23.9 (2.8)	0.919	23.7 (3.2)	23.9 (2.8)	0.513
LDL (mmol/l)	3.64 (1.01)	3.00 (0.95)	<0.001	3.60 (0.98)	3.02 (0.97)	<0.001
HDL (mmol/l)	1.43 (0.41)	1.54 (0.51)	0.082	1.53 (0.48)	1.51 (0.50)	0.791
Triglycerides (mmol/l)	1.25 [0.93–1.70]	0.81[0.63–1.19]	<0.001	1.29 [0.90–1.69]	0.81 [0.64–1.18]	<0.001
Serum creatinine (µmol/l)	92.1 [67–135]	70 [62–81]	<0.001	94.0 [68–132]	69 [62–81]	<0.001
eGFR (ml/min/1.73 m^2^)	76.0 (31.6)	101 (21.5)	<0.001	76.2 (30.9)	100.7 (22.2)	<0.001
Urinary albumin excretion (mg/24 h)	658 [34–1983]	17 [7–532]	<0.001	761 [96–2040]	17 [7–479]	<0.001
Systolic blood pressure (mmHg)	157 (24)	136 (20)	<0.001	159 (24)	136 (19)	<0.001
Diastolic blood pressure (mmHg)	85 (13)	80 (12)	0.001	87 (14)	79 (11)	<0.001
Mean arterial pressure (mmHg)	109 (15)	98 (13)	<0.001	111 (15)	98 (13)	<0.001
RAAS-inhibitors (%)	52	21	<0.001	52	22	<0.001
Other antihypertensive medication (%)	64	29	<0.001	69	28	<0.001
Smoking (never/former/current) (%)	28/19/53	38/17/45	0.234	25/17/58	39/18/43	0.05
MMP-1 (ng/ml)	4.58 [2.78–7.17]	2.70 [1.67–4.71]	<0.001	5.00 [2.74–7.03]	2.73 [1.68–4.68]	<0.001
MMP-2 (ng/ml)	229 [201–273]	185 [162–225]	<0.001	231 [201–281]	185 [161–225]	<0.001
MMP-3 (ng/ml)	21.3 [13.0–28.3]	12.8 [8.6–21.3]	<0.001	23.9 [16.6–29.6]	12.6 [8.5–20.2]	<0.001
MMP-9 (ng/ml)	32.5 [20.4–65.3]	21.7 [14.5–36.9]	<0.001	31.2 [20.7–63.6]	21.8 [14.3–37.0]	<0.001
MMP-10 (pg/ml)	764 [618–1227]	640 [467–930]	0.002	809 [627–1235]	640 [463–916]	<0.001
TIMP-1 (ng/ml)	210 [178–265]	161 [137–204]	<0.001	221 [182–277]	159 [136–204]	<0.001
sVCAM-1 (ng/ml)	1003 [842–1174]	905 [789–1105]	0.03	1038 [936–1239]	891 [789–1096]	<0.001
sICAM-1 (ng/ml)	735 [628–835]	671 [572–814]	0.076	751 [632–882]	670 [569–797]	0.003
Endothelial dysfunction z-score	0.23 (0.92)	−0.08 (1.02)	0.012	0.41 (0.98)	−0.13 (0.97)	<0.001
CRP (mg/l)	1.63 [0.64–3.24]	1.00 [0.42–2.15]	0.008	1.45 [0.60–3.30]	1.03 [0.45–2.18]	0.02
IL-6 (pg/ml)	2.18 [1.53–3.43]	1.52 [0.99–2.34]	<0.001	2.41 [1.78–3.88]	1.45 [1.00–2.22]	<0.001
sPLA2 (µg/ml)	4.40 [2.80–7.00]	4.00 [2.70–6.40]	0.421	4.00 [2.80–6.50]	4.05 [2.70–6.60]	0.821
Low-grade inflammation z-score	0.32 (0.86)	−0.11 (1.02)	<0.001	0.3 (0.96)	−0.11 (0.99)	<0.001

Data are means (SD), medians [inter-quartile range] or percentages, as appropriate

*eGFR* estimated glomerular filtration rate by Chronic Kidney Disease Epidemiology Collaboration (CKD-EPI); *RAAS-inhibitors* renin-angiotensin-aldosterone system inhibitors, including angiotensin converting enzyme inhibitors, angiotensin II receptor blockers and spironolactone; *MMP* matrix metalloproteinase; *TIMP-1* tissue inhibitor of metalloproteinase-1; *sVCAM-1* soluble vascular cell adhesion molecule-1; *sICAM-1* soluble intracellular adhesion molecule-1; *CRP* C-reactive protein; *IL-6* interleukin-6; *sPLA2* secreted phospholipase A2; *Low-grade inflammation z-score* z-score of the average of the z-scores of lnIL-6, lnCRP, sICAM-1, and lnsPLA2; *Endothelial dysfunction z-score* z-score of the average of the z-scores of sICAM-1 and sVCAM-1

### Associations of MMPs and TIMP-1 with incident CVD

Additional file 1: Figure S2 shows the cumulative hazard plots of cardiovascular events according to tertiles of lnMMPs and lnTIMP-1. After adjustment for age, sex, duration of diabetes, HbA1c and nephropathy, higher levels of MMPs 1, 2, 3 and 9, and TIMP-1 were significantly associated with incidence of cardiovascular events, with a hazard ratio (HR) of 1.51 (95% CI 1.20; 1.91) per 1 SD higher lnMMP-1; 1.65 (1.28; 2.13) for lnMMP-2; 1.47 (1.13; 1.92) for lnMMP-3; 1.44 (1.17; 1.78) for lnMMP-9; and 1.87 (1.40; 2.49) for lnTIMP-1, respectively (Table 2, model 1). LnMMP-10 was not significantly associated with incident CVD. After further adjustment for other cardiovascular risk factors, antihypertensive treatment and continuation of antihypertensive treatment at baseline, higher lnMMP-2 [1.49 (1.11; 1.99)] remained significantly associated with the incidence of cardiovascular events (Table 2, model 2). This HR remained significant after further adjustment for markers of LGI and ED (Table 2, models 4 and 5). However, further adjustment for eGFR and lnUAE (Table 2, model 3) decreased the HR from 1.49 (1.11; 1.99) to 1.34 (0.96; 1.85). This decline was explained mainly by the adjustment for eGFR, which decreased the HR to 1.36 (0.98; 1.88), whereas adjustment for lnUAE decreased the HR to 1.46 (1.08; 1.98).

**Table 2:** Associations between baseline plasma lnMMP-1, -2, -3, -9 and -10 and lnTIMP-1 and incident cardiovascular disease (n = 86)

Model	MMP-1			MMP-2			MMP-3			MMP-9			MMP-10			TIMP-1		
	HR	95% CI	p value	HR	95% CI	p value	HR	95% CI	p value	HR	95% CI	p value	HR	95% CI	p value	HR	95% CI	p value
1	1.51	1.20; 1.91	<0.001	1.65	1.28; 2.13	<0.001	1.47	1.13; 1.92	0.004	1.44	1.17; 1.78	0.001	1.20	0.97; 1.50	0.101	1.87	1.40; 2.49	<0.001
2	1.26	1.00; 1.60	0.054	1.49	1.11; 1.99	0.007	1.16	0.88; 1.53	0.308	1.24	0.99; 1.55	0.058	1.03	0.82; 1.29	0.801	1.33	0.95; 1.87	0.100
3	1.23	0.96; 1.59	0.103	1.34	0.96; 1.85	0.085	0.99	0.73; 1.34	0.991	1.21	0.96; 1.52	0.112	0.91	0.71; 1.16	0.440	1.03	0.72; 1.49	0.858
4	1.23	0.97; 1.55	0.094	1.47	1.11; 1.96	0.008	1.15	0.87; 1.52	0.321	1.18	0.93; 1.48	0.173	1.02	0.81; 1.28	0.890	1.23	0.87; 1.75	0.243
5	1.28	1.01; 1.63	0.043	1.48	1.11; 1.98	0.008	1.16	0.88; 1.54	0.284	1.24	1.00; 1.55	0.055	1.03	0.82; 1.29	0.799	1.32	0.94; 1.87	0.111

*HR*, hazard ratio for incident cardiovascular disease per 1 SD increase in lnMMP-1, -2, -3, -9 or -10 or lnTIMP-1, *MMP* matrix metalloproteinase, *TIMP-1* tissue inhibitor of metalloproteinase-1

*Model 1* adjusted for age, sex, HbA1c, nephropathy-no nephropathy status and duration of diabetes

*Model 2* Model 1 + MAP, BMI, smoking status, total cholesterol, use of antihypertensive agents and continuation of medication use at baseline

*Model 3* Model 2 + eGFR and Ln-UAE

*Model 4* Model 2 + inflammatory z-score

*Model 5* Model 2 + endothelial dysfunction z-score

### Associations of MMPs and TIMP-1 with all-cause mortality

Additional file 1: Figure S3 shows the cumulative hazard plots of all-cause mortality according to tertiles of lnMMPs and lnTIMP-1. After adjustment for age, sex, duration of diabetes, HbA1c and nephropathy, higher levels of MMPs 1, 2, 3, 9 and 10, and TIMP-1 were significantly associated with all-cause mortality, with HRs of 1.62 (1.28; 2.06) per 1 SD higher lnMMP-1; 1.93 (1.48; 2.51) for lnMMP-2; 1.82 (1.38; 2.41) for lnMMP-3; 1.39 (1.14; 1.69) for lnMMP-9; 1.33 (1.06; 1.67) for lnMMP-10; and 2.10 (1.55; 2.85) for lnTIMP-1, respectively (Table 3, model 1). After adjustment for other cardiovascular risk factors, antihypertensive treatment and continuation of antihypertensive treatment at baseline, higher lnMMP-1 [1.38 (1.07; 1.78)], lnMMP-2 [1.60 (1.19; 2.15)] and lnMMP-3 [1.39 (1.05; 1.85)] remained significantly associated with all-cause mortality (Table 3, model 2). These associations were independent of eGFR, UAE, LGI and ED (Table 3, models 3–5), except for the association between lnMMP-3 and all-cause mortality, where the HR decreased from 1.39 (1.05; 1.85) to 1.32 (0.95; 1.83) after adjustment for eGFR and lnUAE (Table 3, model 3). This decrease was explained mainly by the adjustment for eGFR, which decreased the HR to 1.32 (0.96; 1.84), whereas adjustment for lnUAE decreased the HR to 1.36 (1.02; 1.82). The associations between MMP-1, MMP-2 and MMP-3 and all-cause mortality did not materially change after mutual adjustments for each of the other MMPs, except for the association between MMP-3 and all-cause mortality, which decreased after adjustment for MMP-1 [to 1.29 (0.96; 1.73)] and MMP-2 [to 1.20 (0.88; 1.62)].

**Table 3:** Associations between baseline plasma lnMMP-1, -2, -3, -9 and -10 and lnTIMP-1 and all-cause mortality (n=83)

Model	MMP-1			MMP-2			MMP-3			MMP-9			MMP-10			TIMP-1		
	HR	95% CI	p va ue	HR	95% CI	p value	HR	95% CI	p value	HR	95% CI	p value	HR	95% CI	p value	HR	95% CI	p value
1	1.62	1.28; 2.06	<0.001	1.93	1.48; 2.51	<0.001	1.82	1.38; 2.41	<0.001	1.39	1.14; 1.69	0.001	1.33	1.06; 1.67	0.015	2.10	1.55; 2.85	<0.001
2	1.38	1.07; 1.78	0.012	1.60	1.19; 2.15	0.002	1.39	1.05; 1.85	0.021	1.18	0.95; 1.46	0.133	1.13	0.90; 1.41	0.313	1.39	0.97; 1.98	0.070
3	1.45	1.10; 1.92	0.008	1.50	1.07; 2.09	0.017	1.32	0.95; 1.83	0.100	1.17	0.93; 1.46	0.179	1.01	0.78; 1.30	0.943	1.15	0.75; 1.74	0.529
4	1.34	1.04; 1.73	0.026	1.57	1.17; 2.10	0.002	1.40	1.06; 1.86	0.019	1.12	0.90; 1.40	0.314	1.12	0.90; 1.41	0.316	1.29	0.89; 1.87	0.173
5	1.47	1.13; 1.92	0.004	1.58	1.17; 2.12	0.002	1.41	1.07; 1.87	0.015	1.18	0.95; 1.45	0.132	1.13	0.90; 1.41	0.307	1.33	0.93; 1.92	0.118

*HR* hazard ratio for all-cause mortality per 1 SD increase in lnMMP-1, -2, -3, -9 or -10 or lnTIMP-1, *MMP* matrix metalloproteinase, *TIMP-1* tissue inhibitor of metalloproteinase-1

*Model 1* adjusted for age, sex, HbA1c, nephropathy-no nephropathy status and duration of diabetes

*Model 2* Model 1 + MAP, BMI, smoking status, total cholesterol, use of antihypertensive agents and continuation of medication use at baseline

*Model 3* Model 2 + eGFR and Ln-UAE

*Model 4* Model 2 + inflammatory z-score

*Model 5* Model 2 + endothelial dysfunction z-score

### Associations of MMPs and TIMP-1 with eGFR, lnUAE, LGI and ED

Cross-sectionally and after full adjustment for cardiovascular risk factors, lnMMP-1, -2, -3, -10 and lnTIMP-1 were significantly and inversely associated with eGFR at baseline (Table 4, model 2). LnMMP-2, -3, -10 and lnTIMP-1 were significantly and positively associated with lnUAE (Table 4, model 2). LGI was significantly associated with lnMMP-1, -9 and lnTIMP-1, and ED was significantly associated with lnMMP-1 and lnTIMP-1 (Table 4, model 2).

**Table 4:** Associations between plasma lnMMP-1, -2, -3, -9 and -10 and lnTIMP-1 and estimated glomerular filtration rate, urinary albumin excretion, low-grade inflammation and endothelial dysfunction

Model	eGFR			Ln-UAE			LGI			ED		
	β	95% CI	p value	β	95% CI	p value	β	95% CI	p value	β	95% CI	p value
MMP-1												
1	−4.15	−6.58; −1.73	0.001	−0.01	−0.13; 0.11	0.912	0.14	0.02; 0.25	0.017	−0.07	−0.18; 0.05	0.249
2	−2.90	−5.22; −0.57	0.015	−0.03	−0.14; 0.08	0.588	0.11	0.00; 0.22	0.045	−0.12	−0.23; −0.01	0.031
MMP-2												
1	−11.9	−14.27; −9.56	<0.001	0.29	0.16; 0.41	<0.001	−0.02	−0.14; 0.10	0.770	0.06	−0.06; 0.18	0.333
2	−10.1	−12.48; −7.64	<0.001	0.19	0.07; 0.32	0.002	0.02	−0.11; 0.15	0.759	0.07	−0.06; 0.19	0.318
MMP-3												
1	−12.6	−15.37; −9.92	<0.001	0.28	0.13; 0.42	<0.001	0.02	−0.12; 0.16	0.779	0.02	−0.12; 0.16	0.792
2	−10.3	−13.05; −7.64	<0.001	0.16	0.02; 0.30	0.022	0.05	−0.09; 0.19	0.513	0.02	−0.12; 0.16	0.818
MMP-9												
1	−0.72	−3.13; 1.70	0.559	0.02	−0.09; 0.14	0.708	0.28	0.18; 0.39	<0.001	0.05	−0.06; 0.16	0.347
2	−0.14	−2.56; 2.29	0.912	0.02	−0.10; 0.13	0.781	0.22	0.11; 0.33	<0.001	−0.03	−0.15; 0.08	0.580
MMP-10												
1	−6.45	−8.70; −4.19	<0.001	0.08	−0.03; 0.20	0.146	0.14	0.03; 0.25	0.010	0.13	0.03; 0.24	0.015
2	−6.18	−8.32; −4.03	<0.001	0.11	0.00; 0.21	0.045	0.09	−0.02; 0.19	0.104	0.09	−0.02; 0.19	0.110
TIMP-1												
1	−10.2	−12.65; −7.82	<0.001	0.26	0.14; 0.39	<0.001	0.30	0.19; 0.42	<0.001	0.23	0.11; 0.35	<0.001
2	−8.13	−10.58; −5.68	<0.001	0.17	0.05; 0.29	0.005	0.33	0.21; 0.45	<0.001	0.22	0.10; 0.34	<0.001

The standardized regression coefficient β represents the difference in eGFR (in ml/min/1.73 m^2^), lnUAE (in mg/24 h), LGI (in SD) or ED (in SD) per 1 SD increase in lnMMP-1, -2, -3, -9, and -10 and lnTIMP-1

*MMP* matrix metalloproteinase, *TIMP-1* tissue inhibitor of metalloproteinase-1

*Model 1* adjusted for age, sex, HbA1c, nephropathy-no nephropathy status and duration of diabetes

*Model 2* Model 1 + MAP, BMI, smoking status, total cholesterol, use of antihypertensive agents and continuation of medication use at baseline

### MMP-2 and -3 and eGFR decline during follow-up

The significant associations between lnMMP-2 and incident CVD and between lnMMP-3 and all-cause mortality were attenuated after adjustment for eGFR. To further investigate the role of eGFR in the association between plasma levels of MMPs and TIMP-1 on the one hand and incident CVD and all-cause mortality on the other, we investigated to what extent baseline MMPs and TIMP-1 levels were associated with changes in eGFR during follow-up. In addition, we investigated to what extent *changes* in eGFR (in addition to baseline eGFR) explained the associations between plasma markers and outcome measures when added to model 2. Follow-up data on eGFR, during follow-up measurements in 2003 [20], were available in 317/337 patients; 20 patients were excluded because they underwent kidney transplantation during follow-up and no data were available on eGFR pretransplantation (n = 14) or because no follow-up eGFR measurement was available (n = 6). Median follow-up duration was 9.9 years [9.1; 10.5]. The median decrease in eGFR was −1.67 ml/min/1.73 m^2^/year (interquartile range −3.18 to −0.64). After adjustment for age, sex, duration of diabetes, HbA1c, smoking, MAP, BMI, total cholesterol, antihypertensive medication and eGFR at baseline, lnMMP-2 and -3 (per 1 SD increase) were associated with decline in eGFR of −0.24 (95% CI −0.60; 0.12) and of −0.55 (−0.94; −0.16) ml/min/1.73 m^2^/year, respectively. Additional file 1: Figure S4 shows the decrease in eGFR per year according to tertiles of all MMPs and TIMP-1 after adjustment for the potential confounders used in model 2 plus baseline eGFR. The decline in eGFR was significantly greater in patients with nephropathy compared to those without (p < 0.001).

In the 317 patients with available data on eGFR during follow-up, the HR for developing CVD per 1 SD higher lnMMP-2 decreased from 1.60 (1.17; 2.18) to 1.40 (1.00; 1.96) after additional adjustment for baseline eGFR. Further adjustment for the decline in eGFR further decreased the HR to 1.36 (0.96; 1.92). This indicates that MMP-2-associated decrease of eGFR may partly explain the association between plasma MMP-2 and incident CVD. Next, in these 317 patients, the HR for all-cause mortality per 1 SD increase in lnMMP-3 decreased from 2.04 (1.45; 2.88) to 1.80 (1.22; 2.65) after additional adjustment for baseline eGFR. Further adjustment for the decrease in eGFR did not materially change the HR [1.79 (1.22; 2.62)].

### Additional analyses

Stratified analyses showed that, after full adjustment for cardiovascular risk factors (model 2), one SD higher lnTIMP-1 was associated with an increase in all-cause mortality in patients without [HR 3.65 (1.49; 8.92)], but not in those with nephropathy at baseline [HR 1.15 (0.75; 1.76)] (p for interaction was 0.009). In addition, after full adjustment for cardiovascular risk factors (model 2), one SD higher lnMMP-2 was associated with an increased HR for all-cause mortality in men [HR 1.99 (1.34; 2.95)], but not in women [0.85 (0.49; 1.46)] (p for interaction was 0.025).

Associations between plasma lnMMPs and lnTIMP-1 on the one hand and eGFR, lnUAE, LGI and ED on the other differed significantly between patients with and without nephropathy (Additional file 1: Tables S3, S4). The associations between plasma lnMMPs and lnTIMP-1 and eGFR and UAE were generally stronger in patients with diabetic nephropathy. Associations between markers of LGI and ED on the one hand and MMPs and TIMP-1 on the other did not differ consistently between patients with or without nephropathy.

## Discussion

The present study in type 1 diabetic patients, with a median follow-up of 12.3 years, shows significant associations between higher levels of plasma MMP-2 and incident CVD. In addition, higher plasma levels of MMP-1, -2 and -3 were significantly associated with all-cause mortality. These associations were independent of cardiovascular risk factors, LGI and ED (as expressed by z-scores), but partly dependent on eGFR and changes in eGFR. Despite the widespread correlations between the various MMPs and TIMP-1 (Additional file 1: Table S2), we showed specific associations as mentioned previously, which will be further discussed both with regards to current literature, but also on possible pathophysiological mechanisms.

### MMPs, TIMP-1 and cardiovascular disease

Higher MMP-2-levels were independently associated with incident cardiovascular events, which is supported by the few studies available in mainly non-diabetic subjects [12, 28, 29]. In patients with atrial fibrillation, higher baseline plasma MMP-2 levels were independently associated with cardiovascular events or death during a mean follow-up of 28 months [12]. In a Mexican population, a significant association between the specific MMP-2-1575 A/G polymorphism and occurrence of myocardial infarction has been established [28]. Additionally, in patients with ST-elevation myocardial infarction undergoing percutaneous coronary intervention, higher plasma MMP-2-levels at baseline were associated with increased myocardial infarct size and decreased left ventricular function after four months of follow-up [29].

Various mechanisms may contribute to MMP-2-mediated macrovascular disease. We hypothesize that the occurrence of macrovascular disease can be enhanced by increased extracellular MMP-2 activity leading to plaque rupture, thrombus formation and vasoconstriction caused by MMP-2-mediated cleavage of vasoactive peptides. This hypothesis is supported by current literature [4, 30]. Additionally, altered MMP-2 activity may trigger cardiovascular pathology by vascular remodeling, i.e. increased migration of smooth muscle vascular cells to the intima, increased fibrosis and decrease of elastin content [3, 31]. Furthermore, MMP-2 leads to sustained thrombus formation by increased platelet activation, adhesion and aggregation [4].

In our study plasma MMP-1, -3, -9, -10 and TIMP-1-levels were not associated with incident CVD, which is in line with several prospective studies in the general population [32–34] and in patients with rheumatoid arthritis [35]. In contrast, one study in 1127 patients with stable and unstable angina showed significant associations between higher MMP-9 levels and cardiovascular mortality with a HR of 2.41 (1.26; 4.60) in the upper quartile [13]. The discrepancies with our study can possibly be explained by the differences in study population and by the fact that we used a combined endpoint for fatal and non-fatal CVD. For the combined endpoint of cardiovascular death and nonfatal myocardial infarction in the study by Blankenberg et al. [13], the unadjusted HR was 1.30 (1.11; 1.52, p < 0.002) with increasing MMP-9 quartiles, but it is not clear whether or not this remained significant after adjustment for potential confounders. In our study we did not show an association between TIMP-1-levels and occurrence of CVD. This is in contrast to a recent study which showed a significant association between plasma TIMP-1-levels and recurrent cardiovascular events in patients suffering from previous myocardial infarction or unstable angina 3–36 months before enrollment [14]. This discrepancy might be explained by the difference in study populations, as in our study patients with prior CVD were excluded. In addition, in our study more adjustments for potential confounders were made compared with the latter two studies.

### MMPs, TIMP-1 and all-cause mortality

Baseline MMP-1, -2 and -3-levels were significantly associated with all-cause mortality during follow-up. Stratification according to sex showed that the association between MMP-2 and all-cause mortality was restricted to men. However, the present study can neither explain this sex interaction nor can it exclude the possibility of a chance finding. Thus far, no prospective studies have been published regarding the association between plasma MMPs and all-cause mortality in type 1 diabetic patients. In different study populations higher circulating levels of MMP-1 [15], MMP-2 [12, 16, 17] and MMP-3 [18, 19] were strongly associated with all-cause mortality in patients with colorectal cancer [15], myocardial infarction [16, 18] or heart failure [17, 19]. MMP-1 is thought to be involved in cancer progression and invasion [15], whereas the mechanism by which MMP-3 contributed was not clarified [18, 19].

Plasma TIMP-1 levels did not show a significant association with all-cause mortality in the total study group and in patients *with* nephropathy at baseline. In contrast, plasma TIMP-1 was positively associated with all-cause mortality in patients *without* nephropathy at baseline. However, the observed interaction between TIMP-1 and nephropathy-status should be interpreted with caution as we cannot exclude the play of chance. Previous studies on the association between plasma TIMP-1 and mortality have been conflicting, both no [36] and positive associations [34, 37, 38] have been described. Positive associations between TIMP-1 and mortality were encountered in the general population [34], patients who underwent coronary angiography [37] and in patients with heart failure [38]. These diverse results do not allow us to draw a solid conclusion on the association between TIMP-1 and all-cause mortality for all study populations. However, in type 1 diabetes, the present study is the first to report on this association.

Certain genetic polymorphisms coding for MMP-1 and MMP-3 have been associated with all-cause mortality [39]. The MMP-1 2G/2G polymorphism has been associated with increased mortality in a 3-year follow-up study in 99 hemodialysis patients and 133 matched controls, HR 2.96 (1.29–6.80). In this study, the MMP-3 6A/6A polymorphism was non-significantly associated with all-cause mortality, HR 3.01 (0.88–10.3) and combined homozygosity of MMP-1 and MMP-3 polymorphisms lead to a HR of 4.69 (1.72–12.8). It is unknown, however, how these polymorphisms influence MMP expression and/or activity and the underlying mechanism of this association thus remains to be further investigated.

### Potential mediation through eGFR

The association between plasma MMP-2 and CVD was attenuated after adjustment for baseline eGFR, which explained ~10% of the association. The HR decreased further after adjustment for decline in eGFR during follow-up. This may indicate that eGFR and decline in eGFR play a mediating role in the association between MMP-2 and CVD. In support, MMP-2 has been shown to induce complex alterations characterizing renal tubular epithelial-mesenchymal transformation leading to increased renal fibrosis [40], which could lead to decreased renal function. Higher plasma MMP-2 levels may also be the result of decreased renal function. However, MMP-2 levels are only minimally dependent on renal clearance due to the molecular weight of MMP-2, which is higher than that of albumin.

The association between MMP-3 and all-cause mortality was also attenuated by eGFR and this explained ~5% of the association. Although MMP-3 was significantly associated with eGFR decline during follow-up, the association between MMP-3 and mortality did not materially change after adjustment for eGFR decline. This makes a mediating role for eGFR in the association between MMP-3 and all-cause mortality less likely. Taken together, these results show that increased plasma levels of MMP-2 and -3, possible due to, but to a greater extent independent of, decreased eGFR are positively associated with CVD and all-cause mortality, respectively.

### Limitations

There are several limitations to our study. Firstly, plasma levels of MMP-1, -2, -3, -9, -10 and of TIMP-1 were only determined at baseline. Changes in plasma levels over time, revealing more details about outcome measures, are not known. Secondly, we do not know to what extent plasma levels reflect the local pathological situation at tissue level. Thirdly, we only determined five different MMPs and one of the four known TIMPs, considered to be the most important MMPs and TIMP to contribute to our outcome measures, but we cannot exclude that other MMPs and TIMPs are involved. Fourthly, the non-significant associations between the MMPs/TIMP-1 and outcome measures could be due to lack of statistical power, because of the relatively small number of events. Patients who died during follow-up were classified as cardiovascular unless another cause was established and this could have biased our results towards the null, resulting in underestimation of associations. Finally, the associations between MMPs and TIMP-1 with eGFR changes over time might be weakened by excluding those with renal transplantation during follow-up. Besides these limitations, the current study, with extensive adjustments for potential confounders and mediators, provides additional and consistent information on ECM remodeling by MMPs and TIMP-1 preceding incident CVD and all-cause mortality in a large cohort of patients with type 1 diabetes. These results are generalizable to normo- and macroalbuminuric type 1 diabetic patients. Although microalbuminuric patients were not included in the current study, we have no reason to expect different associations in this group.

## Conclusions

In patients with type 1 diabetes followed for a median of 12 years, higher plasma MMP-2 levels are associated with incident CVD and higher plasma MMP-1, MMP-2, MMP-3 are associated with all-cause mortality. These associations are independent of cardiovascular risk factors, low-grade inflammation and endothelial dysfunction. However, baseline eGFR and decline in eGFR during follow-up attenuated the association between MMP-2 and CVD as well as the association between MMP-3 and all-cause mortality; eGFR may thus, in part, mediate these associations.

## Additional file

**Additional file 1.** Additional figures and tables.

## Abbreviations

CVD: cardiovascular disease
ED: endothelial dysfunction
eGFR: estimated glomerular filtration rate
LGI: low-grade inflammation
MMP: matrix metalloproteinase
TIMP: tissue inhibitor of metalloproteinase
UAE: urinary albumin excretion
