# Supplementary material for: Plasma matrix metalloproteinases are associated with incident cardiovascular disease and all-cause mortality in patients with type 1 diabetes: a 12-year follow-up study
Source: Cardiovasc Diabetol. 2017 Apr 26;16:55. doi: 10.1186/s12933-017-0539-1 (PMC5405549; doi:10.1186/s12933-017-0539-1)
Supplement: Supplementary file 1 — Additional file 1. Additional figures and tables. [file 12933_2017_539_MOESM1_ESM.docx]

**Additional file 1: Figure S1.Overview of study population selection**

Some patients suffered from a non-fatal as well as a fatal CVD event. The first event was used in the analyses.

Cardiovascular disease (CVD), End stage renal disease (ESRD)


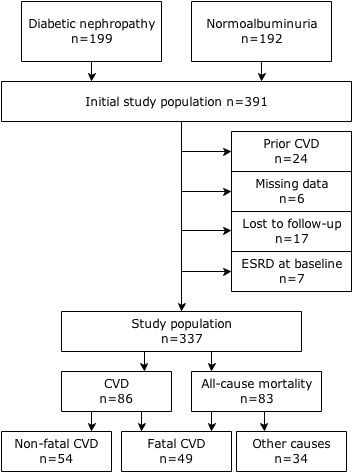


**Additional file 1: Figure S2. Cumulative hazard plots of cardiovascular events according to tertiles of lnMMP1, -2, -3, -9 and -10, and lnTIMP-1**


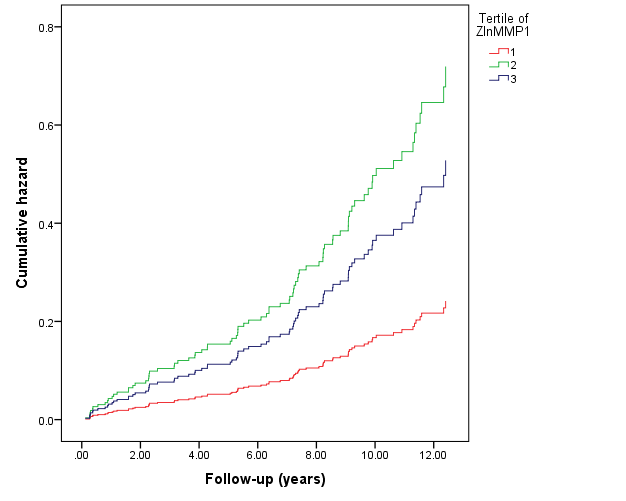

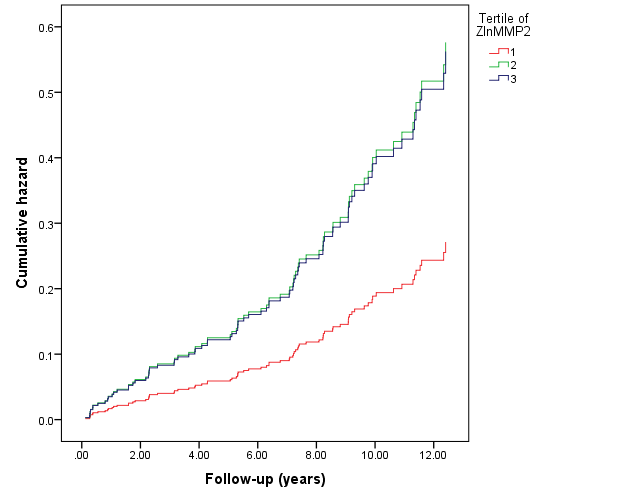

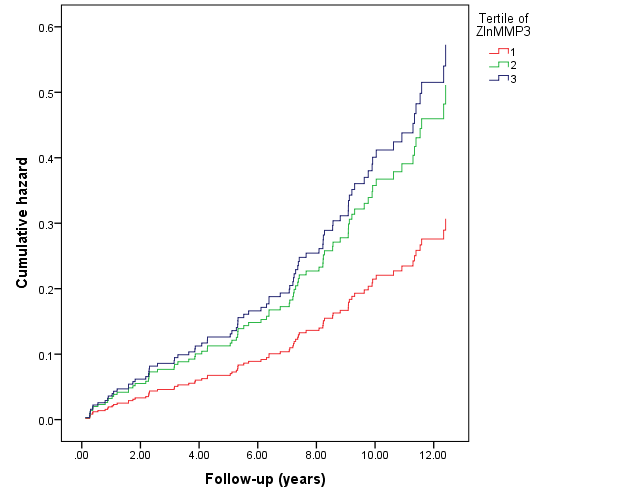


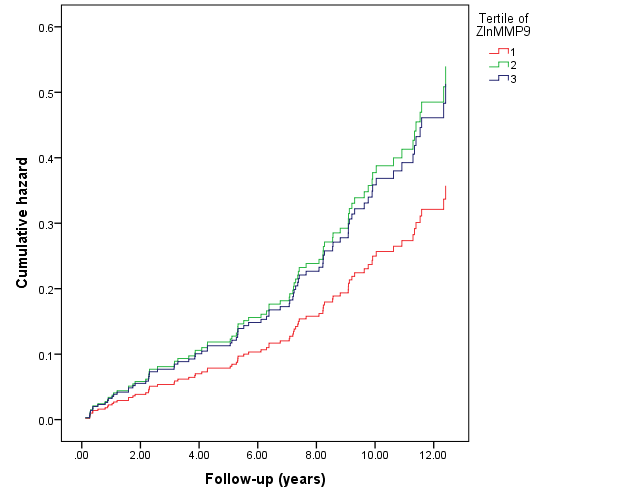

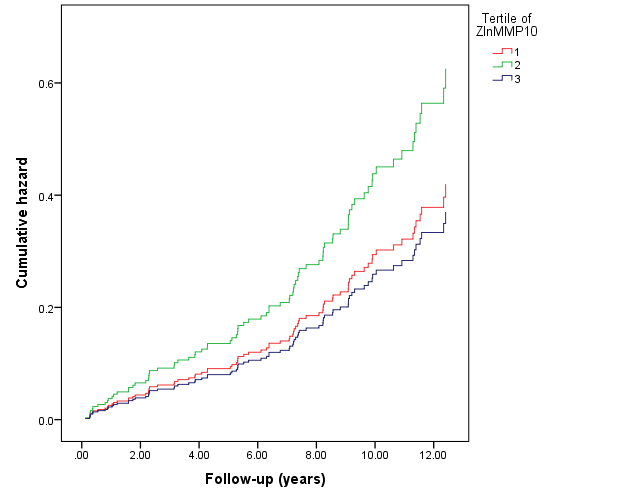

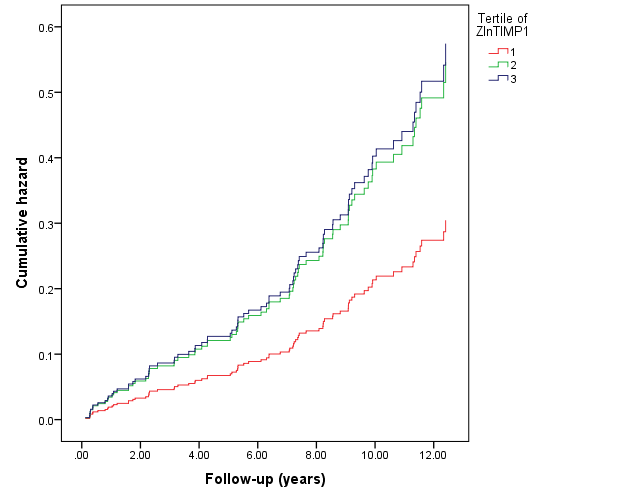


**Additional file 1: Figure S3. Cumulative hazard plots of all-cause mortality according to tertiles of lnMMP1, -2, -3, -9 and -10, and lnTIMP-1**


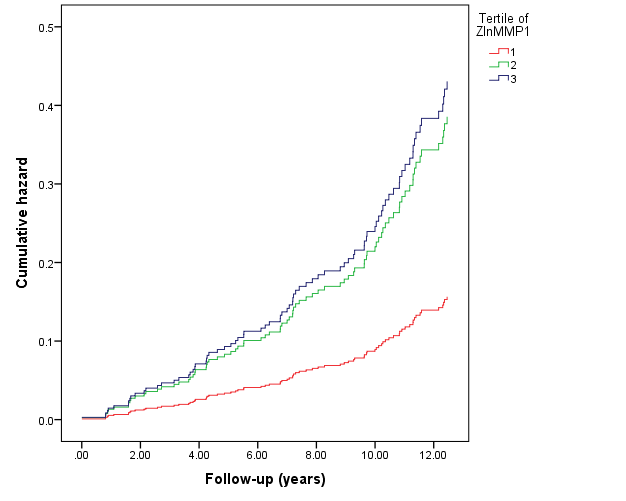

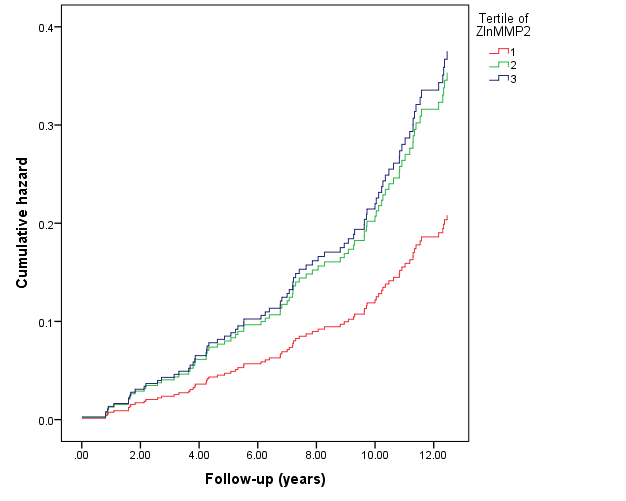

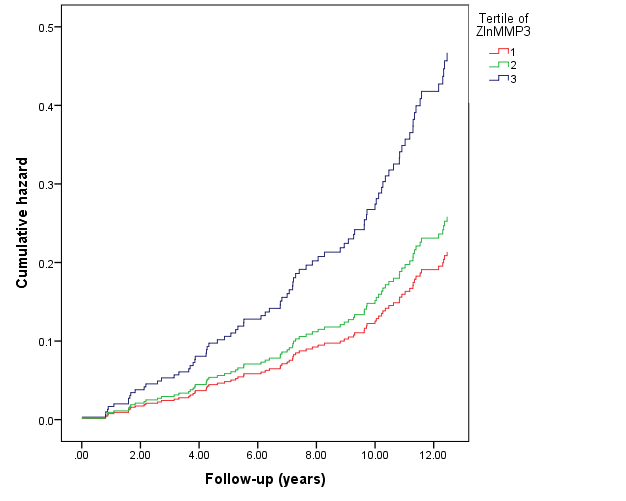


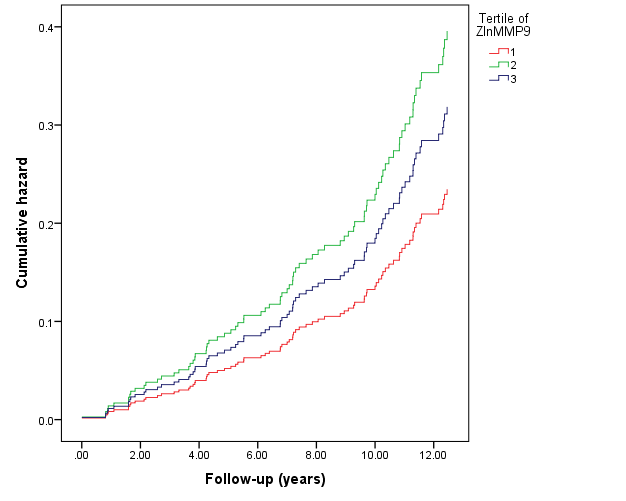

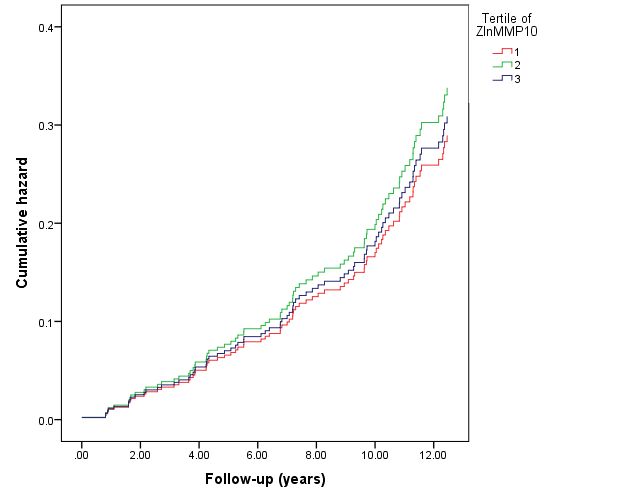

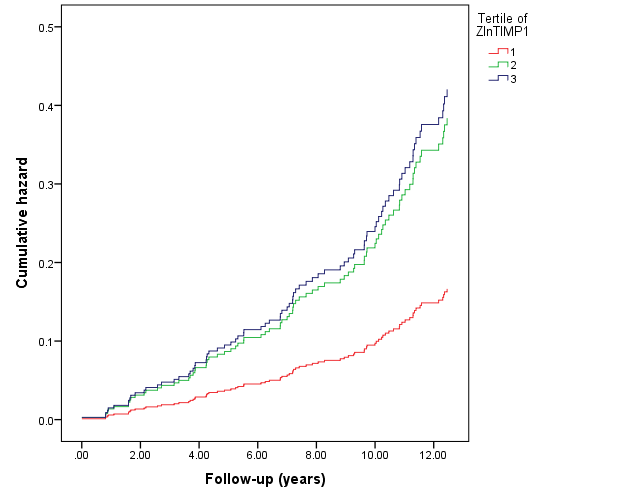


**Additional file 1: Figure S4. Decrease in eGFR (ml/min/1.73m^2^) per year according to tertiles of MMPs and TIMP-1 adjusted for sex, age, duration of diabetes, HbA1c, nephropathy-no nephropathy status, BMI, MAP, smoking status, antihypertensive treatment, continuation of antihypertensive treatment at baseline and baseline eGFR**

**
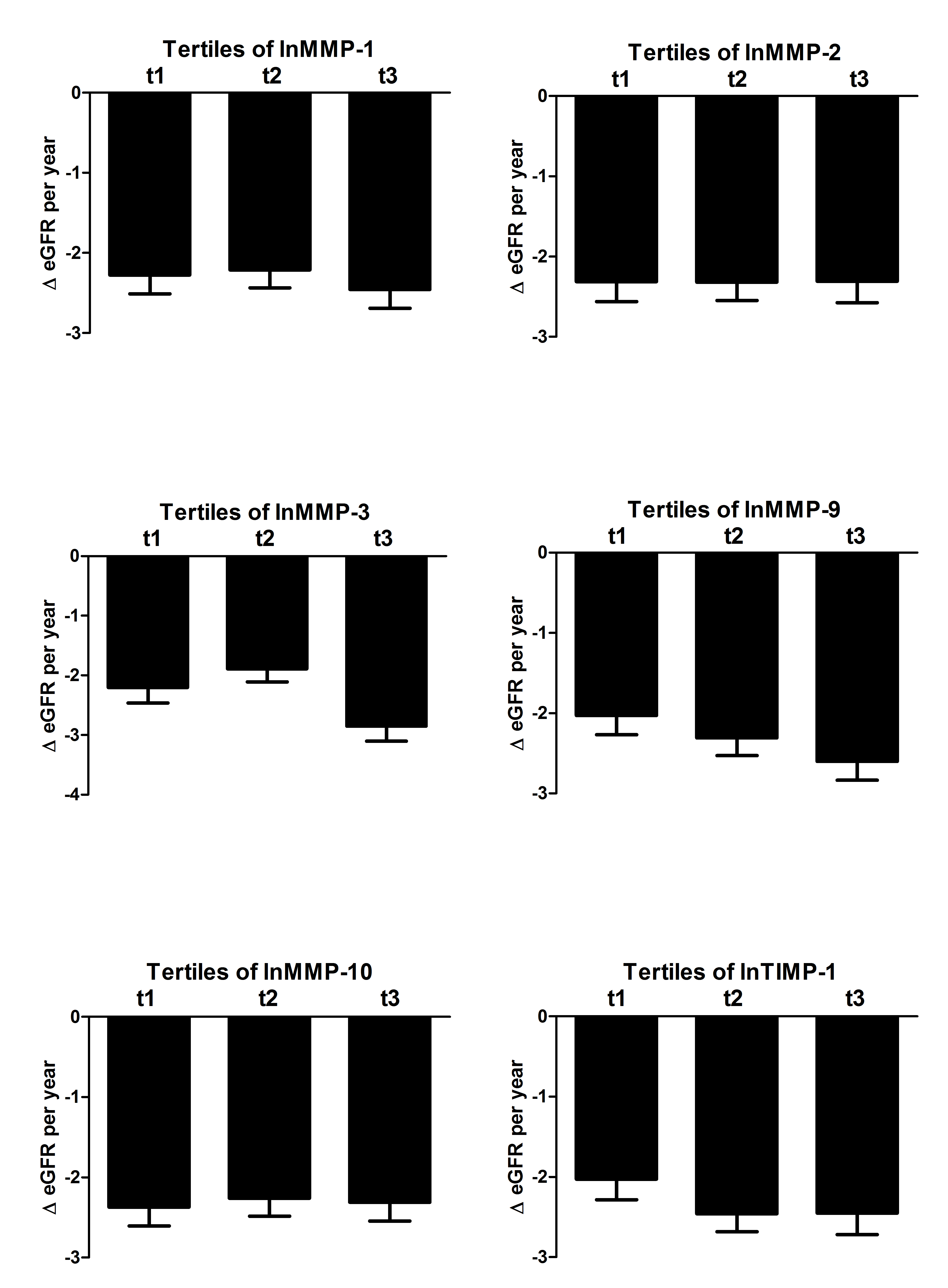
**

**Additional file 1: Table S1. Baseline characteristics according to nephropathy-no nephropathy status**

|  | Nephropathy  n=170 | No nephropathy  n=167 | | p-value |
| --- | --- | --- | --- | --- |
| Age (yrs) | 40.4 (9,5) | | 42.6 (9.7) | 0.035 |
| Sex: male/female (%) | 62/38 | | 59/41 | 0.641 |
| Duration of diabetes (yrs) | 27.9 (8.1) | | 27.6 (8.0) | 0.690 |
| HbA1c (%)  HbA1c (mmol/mol) | 9.5 (1.5)  80.5 (16.9) | | 8.5 (1.1)  69.8 (12.1) | <0.001  <0.001 |
| Retinopathy (no/simplex/proliferative) (%)  BMI (kg/m^2^) | 0/32/68  24.1 (3.3) | | 35/54/11  23.7 (2.5) | <0.001  0.187 |
| LDL (mmol/l)  HDL (mmol/l)  Triglycerides (mmol/l)  Serum creatinine (µmol/l)  eGFR (ml/min/1.73m^2^)  Urinary albumin excretion (mg/24h)  Systolic blood pressure (mmHg)  Diastolic blood pressure (mmHg)  Mean arterial pressure (mmHg)  RAAS-inhibitors (%)  Other antihypertensive medication (%)  Smoking (never/former/current) (%)  MMP-1 (ng/ml)  MMP-2 (ng/ml)  MMP-3 (ng/ml)  MMP-9 (ng/ml) | 3.48 (1.03)  1.48 (0.55)  1.21 [0.85-1.66]  98.5 [80-128]  72.4 (28.5)  823 [360-2050]  151 (23)  86 (12)  108 (14)  54  66  32/17/51  4.08 [2.51-6.89]  230 [194-266]  20.1 [12.6-28.7]  31.1 [21.0-56.0] | | 2.84 (0.87)  1.54 (0.43)  0.77 [0.57-0.95]  76 [69-83]  97.3 (13.3)  8 [5-13]  132 (18)  76 (10)  94 (11)  4  9  39/19/42  2.21 [1.43-3.49]  180 [156-200]  10.6 [7.8-16.6]  18.5 [13.2-30.5] | <0.001  0.264  <0.001  <0.001  <0.001  <0.001  <0.001  <0.001  <0.001  <0.001  <0.001  0.262  <0.001  <0.001  <0.001  <0.001 |
| MMP-10 (pg/ml)  TIMP-1 (ng/ml)  sVCAM-1 (ng/ml)  sICAM-1 (ng/ml)  Endothelial dysfunction z-score  CRP (mg/l)  IL-6 (pg/ml)  sPLA2 (µg/ml)  Low-grade inflammation z-score | 785 [525-1093]  216 [174-264]  1018 [860-1204]  711 [586-848]  0.23 (0.82)  1.22 [0.59-2.94]  2.06 [1.25-3.38]  4.40 [2.70-6.73]  0.13 (0.64) | | 612 [443-873]  149 [129-174]  880 [759-994]  672 [574-776]  -0.23 (0.67)  0.92 [0.34-2.06]  1.40 [0.93-2.08]  4.00 [2.80-6.10]  -0.14 (0.67) | 0.002  <0.001  <0.001  0.063  <0.001  0.720  0.743  0.441  <0.001 |

Data are means (SD), medians [inter-quartile range] or percentages, as appropriate. eGFR, estimated glomerular filtration rate by Chronic Kidney Disease Epidemiology Collaboration (CKD-EPI); RAAS-inhibitors, renin-angiotensin-aldosterone system inhibitors, including angiotensin converting enzyme inhibitors, angiotensin II receptor blockers and spironolactone; MMP, matrix metalloproteinase; TIMP-1, tissue inhibitor of metalloproteinase-1; sVCAM-1, soluble vascular cell adhesion molecule-1; sICAM-1, soluble intracellular adhesion molecule-1; CRP, C-reactive protein; IL-6, interleukin-6; sPLA2, secreted phospholipase A2; Low-grade inflammation z-score, z-score of the average of the z-scores of lnIL-6, lnCRP, sICAM-1, and lnsPLA2; Endothelial dysfunction z-score, z-score of the average of the z-scores of sICAM-1 and sVCAM-1.

**Additional file 1: Table S2. Correlations between plasma MMP-1, -2, -3, -9 and -10, and TIMP-1 (n=337)**

|  | MMP-1 | |  | MMP-2 | |  | MMP-3 | |  | MMP-9 | |  | MMP-10 | |  | TIMP-1 | |
| --- | --- | --- | --- | --- | --- | --- | --- | --- | --- | --- | --- | --- | --- | --- | --- | --- | --- |
|  | r | p-value |  | r | p-value |  | r | p-value |  | r | p-value |  | r | p-value |  | r | p-value |
| MMP-1 | 1.00 | - |  | 0.35 | <0.001 |  | 0.33 | <0.001 |  | 0.28 | <0.001 |  | 0.11 | 0.05 |  | 0.51 | <0.001 |
| MMP-2 | 0.35 | <0.001 |  | 1.00 | - |  | 0.53 | <0.001 |  | 0.05 | 0.346 |  | 0.28 | <0.001 |  | 0.62 | <0.001 |
| MMP-3 | 0.33 | <0.001 |  | 0.53 | <0.001 |  | 1.00 | - |  | 0.08 | 0.164 |  | 0.31 | <0.001 |  | 0.55 | <0.001 |
| MMP-9 | 0.28 | <0.001 |  | 0.05 | 0.346 |  | 0.08 | 0.164 |  | 1.00 | - |  | 0.07 | 0.197 |  | 0.25 | <0.001 |
| MMP-10 | 0.11 | 0.05 |  | 0.28 | <0.001 |  | 0.31 | <0.001 |  | 0.07 | 0.197 |  | 1.00 | - |  | 0.29 | <0.001 |
| TIMP-1 | 0.51 | <0.001 |  | 0.62 | <0.001 |  | 0.55 | <0.001 |  | 0.25 | <0.001 |  | 0.29 | <0.001 |  | 1.00 | - |

r, Pearson correlation

**Additional file 1: Table S3. Associations between plasma lnMMP-1, -2, -3, -9 and -10, and lnTIMP-1 and estimated glomerular filtration rate, urinary albumin excretion, low-grade inflammation and endothelial dysfunction in patients with diabetic nephropathy (n=170)**

|  |  |  | eGFR |  |  |  | Ln-UAE |  |  |  | LGI |  |  |  | ED |  |
| --- | --- | --- | --- | --- | --- | --- | --- | --- | --- | --- | --- | --- | --- | --- | --- | --- |
|  | model | β | 95%CI | p-value |  | β | 95%CI | p-value |  | β | 95%CI | p-value |  | β | 95%CI | p-value |
| MMP-1 | 1 | -7.80 | -12.13;-3.47 | <0.001 |  | 0.00 | -0.19;0.20 | 0.986 |  | 0.15 | 0.01;0.30 | 0.043 |  | -0.02 | -0.18;0.14 | 0.810 |
|  | 2 | -4.36 | -8.41;-0.31 | 0.035 |  | -0.07 | -0.24;0.11 | 0.464 |  | 0.10 | -0.05;0.26 | 0.190 |  | -0.11 | -0.28;0.06 | 0.205 |
| MMP-2 | 1 | -17.5 | -21.1;-14.0 | <0.001 |  | 0.41 | 0.22;0.59 | <0.001 |  | 0.06 | -0.09;0.20 | 0.468 |  | 0.15 | -0.01;0.31 | 0.069 |
|  | 2 | -13.8 | -17.79;-9.88 | <0.001 |  | 0.22 | 0.03;0.42 | 0.024 |  | 0.06 | -0.11;0.23 | 0.477 |  | 0.12 | -0.06;0.31 | 0.192 |
| MMP-3 | 1 | -20.6 | -25.2;-15.9 | <0.001 |  | 0.44 | 0.21;0.68 | <0.001 |  | -0.02 | -0.20;0.17 | 0.875 |  | 0.02 | -0.18;0.23 | 0.872 |
|  | 2 | -15.6 | -20.5;-10.8 | <0.001 |  | 0.19 | -0.04;0.43 | 0.108 |  | -0.04 | -0.24;0.17 | 0.708 |  | -0.04 | -0.26;0.19 | 0.743 |
| MMP-9 | 1 | -2.17 | -6.57;2.22 | 0.330 |  | 0.05 | -0.14;0.25 | 0.582 |  | 0.25 | 0.11;0.39 | 0.001 |  | -0.01 | -0.17;0.15 | 0.919 |
|  | 2 | 0.12 | -4.06;4.31 | 0.954 |  | 0.01 | -0.18;0.19 | 0.938 |  | 0.18 | 0.02;0.33 | 0.026 |  | -0.11 | -0.29;0.06 | 0.199 |
| MMP-10 | 1 | -11.9 | -16.02;-7.72 | <0.001 |  | 0.07 | -0.13;0.27 | 0.491 |  | 0.12 | -0.04;0.27 | 0.131 |  | 0.15 | -0.01;0.32 | 0.066 |
|  | 2 | -9.65 | -13.49;-5.81 | <0.001 |  | 0.04 | -0.14;0.22 | 0.636 |  | 0.05 | -0.10;0.21 | 0.504 |  | 0.09 | -0.09;0.26 | 0.321 |
| TIMP-1 | 1 | -21.6 | -25.8;-17.4 | <0.001 |  | 0.62 | 0.41;0.83 | <0.001 |  | 0.39 | 0.22;0.55 | <0.001 |  | 0.45 | 0.27;0.63 | <0.001 |
|  | 2 | -17.7 | -22.7;-12.6 | <0.001 |  | 0.46 | 0.22;0.70 | <0.001 |  | 0.47 | 0.26;0.67 | <0.001 |  | 0.52 | 0.29;0.74 | <0.001 |

The standardized regression coefficient β represents the difference in eGFR (in ml/min/1.73m^2^), lnUAE (in mg/24h), LGI (in SD) or ED (in SD)

per 1 SD increase in lnMMP-1, -2, -3, -9, and -10 and lnTIMP-1. MMP, matrix metalloproteinase; TIMP-1, tissue inhibitor of metalloproteinase-1.

| Model 1 | Adjusted for age, sex, HbA1c and duration of diabetes |
| --- | --- |
| Model 2 | Model 1 + MAP, BMI, smoking status, total cholesterol, use of antihypertensive agents and continuation of medication use at baseline |

**Additional file 1: Table S4. Associations between plasma lnMMP-1, -2, -3, -9 and -10 and lnTIMP-1 and estimated glomerular filtration rate, urinary albumin excretion, low-grade inflammation and endothelial dysfunction in patients with normoalbuminuria (n=167)**

|  |  |  | eGFR |  |  |  | Ln-UAE |  |  |  | LGI |  |  |  | ED |  |
| --- | --- | --- | --- | --- | --- | --- | --- | --- | --- | --- | --- | --- | --- | --- | --- | --- |
|  | model | β | 95%CI | p-value |  | β | 95%CI | p-value |  | β | 95%CI | p-value |  | β | 95%CI | p-value |
| MMP-1 | 1 | 0.29 | -1.13;1.71 | 0.683 |  | -0.08 | -0.20;0.05 | 0.216 |  | 0.10 | -0.08;0.27 | 0.274 |  | -0.16 | -0.31;-0.01 | 0.043 |
|  | 2 | -0.13 | -1.50;1.24 | 0.853 |  | -0.07 | -0.20;0.05 | 0.253 |  | 0.10 | -0.07;0.27 | 0.232 |  | -0.20 | -0.35;-0.05 | 0.010 |
| MMP-2 | 1 | -0.07 | -1.82;1.67 | 0.934 |  | 0.09 | -0.07;0.24 | 0.271 |  | -0.19 | -0.40;0.03 | 0.088 |  | -0.14 | -0.32;0.05 | 0.156 |
|  | 2 | 0.20 | -1.47;1.86 | 0.817 |  | 0.07 | -0.08;0.22 | 0.375 |  | -0.17 | -0.37;0.04 | 0.105 |  | -0.11 | -0.29;0.08 | 0.271 |
| MMP-3 | 1 | -2.16 | -3.83;-0.49 | 0.012 |  | 0.10 | -0.05;0.25 | 0.172 |  | 0.10 | -0.11;0.31 | 0.339 |  | 0.04 | -0.14;0.23 | 0.663 |
|  | 2 | -2.05 | -3.63;-0.48 | 0.011 |  | 0.09 | -0.05;0.24 | 0.209 |  | 0.11 | -0.09;0.30 | 0.291 |  | 0.05 | -0.13;0.23 | 0.590 |
| MMP-9 | 1 | 1.22 | -0.12;2.56 | 0.073 |  | -0.04 | -0.15;0.08 | 0.549 |  | 0.33 | 0.17;0.49 | <0.001 |  | 0.12 | -0.02;0.27 | 0.095 |
|  | 2 | 0.95 | -0.42;2.32 | 0.172 |  | -0.02 | -0.14;0.11 | 0.799 |  | 0.28 | 0.11;0.44 | 0.001 |  | 0.06 | -0.10;0.21 | 0.483 |
| MMP-10 | 1 | -0.39 | -1.64;0.85 | 0.536 |  | 0.13 | 0.02;0.24 | 0.020 |  | 0.18 | 0.03;0.33 | 0.018 |  | 0.13 | -0.01;0.26 | 0.064 |
|  | 2 | -0.54 | -1.75;0.68 | 0.385 |  | 0.16 | 0.05;0.27 | 0.004 |  | 0.10 | -0.05;0.25 | 0.204 |  | 0.09 | -0.05;0.22 | 0.223 |
| TIMP-1 | 1 | -0.51 | -1.85;0.83 | 0.455 |  | -0.06 | -0.17;0.06 | 0.339 |  | 0.22 | 0.06;0.38 | 0.008 |  | 0.03 | -0.12;0.17 | 0.690 |
|  | 2 | -0.47 | -1.74;0.80 | 0.463 |  | -0.06 | -0.18;0.05 | 0.285 |  | 0.22 | 0.07;0.37 | 0.005 |  | 0.04 | -0.11;0.18 | 0.595 |

The standardized regression coefficient β represents the difference in eGFR (in ml/min/1.73m^2^), lnUAE (in mg/24h), LGI (in SD) or ED (in SD)

per 1 SD increase in lnMMP-1, -2, -3, -9, and -10 and lnTIMP-1. MMP, matrix metalloproteinase; TIMP-1, tissue inhibitor of metalloproteinase-1.

| Model 1 | Adjusted for age, sex, HbA1c and duration of diabetes |
| --- | --- |
| Model 2 | Model 1 + MAP, BMI, smoking status, total cholesterol, use of antihypertensive agents and continuation of medication use at baseline |
